# Preterm Neonates with Respiratory Distress Syndrome: Ventilator-Induced Lung Injury and Oxidative Stress

**DOI:** 10.1155/2018/6963754

**Published:** 2018-04-23

**Authors:** Clarissa Gutiérrez Carvalho, Renato Soibelmann Procianoy, Eurico Camargo Neto, Rita C. Silveira

**Affiliations:** ^1^Department of Pediatrics, Universidade Federal do Rio Grande do Sul, Porto Alegre, RS, Brazil; ^2^Newborn Section, Hospital de Clínicas de Porto Alegre, Porto Alegre, RS, Brazil

## Abstract

Ventilator-induced lung injury is well recognized, and appropriate arterial saturation target is unknown, so gentle modes of ventilation and minimizing oxidative stress have been well studied. Our objective was to analyze any association between the oxygen levels at blood sampling and plasma levels of the interleukins IL-6, IL-1*β*, IL-10, and IL-8 and TNF-*α* in preterm newborns under mechanical ventilation (MV) in their first two days. *Methods*. Prospective cohort including neonates with severe respiratory distress. Blood samples were collected right before and 2 hours after invasive MV. For analysis purposes, newborns were separated according to oxygen requirement: low oxygen (≤30%) and high oxygen (>30%) groups. Interleukins were measured using a commercially available kit. *Results*. 20 neonates (gestational age 32.2 ± 3 weeks) were evaluated. Median O_2_ saturation levels pre-MV were not different in both oxygen groups. In the high oxygen group, IL-6, IL-8, and TNF-*α* plasma levels increased significantly after two hours under MV. *Conclusions*. Despite the small sample studied, data showed that there is a relationship between VILI, proinflammatory cytokines, and oxygen-induced lung injury, but a study considering oxidative marker measurements is needed. It seems that less oxygen may keep safer saturation targets playing a less harmful role.

## 1. Introduction

It is well recognized that in preterm neonates, the need for invasive mechanical ventilation (MV) is associated with the so-called ventilator-induced lung injury (VILI); thus, lung protection strategies have been intensely studied in the past 20 years. Research has focused on gentle modes of ventilation as well as in reducing oxidative stress.

Superoxide, hydrogen peroxide, and perhydroxyl radicals cause oxygen-induced lung injury, and the premature infant is notably susceptible to free radical-induced injury because antioxidant systems develop late during the gestation. Excessive hyperoxia can lead to lung inflammation, diffuse alveolar injury, progressive pulmonary damage, and death [1].

The suitable arterial saturation target in preterm neonates is unknown, but exposing them to a high concentration of oxygen is related to increased risks of retinopathy of prematurity and bronchopulmonary dysplasia (BPD). Recently published large multicenter trials have studied this issue [2].

In this pilot study, our objective was to analyze any association between the oxygen levels at blood sampling and plasma levels of interleukin- (IL-) 6, IL-1*β*, IL-10, IL-8, and tumor necrosis factor- (TNF-) *α* in preterm infants under MV in their first two days of life.

## 2. Methods

This prospective observational study included preterm infants ranging from 28 to 35 weeks of gestational age (GA) submitted to intubation and MV in the first 48 hours of life who were admitted to the newborn section of Hospital de Clínicas de Porto Alegre (HCPA), a tertiary referral medical center located in Southern Brazil. The Ethics Committee of HCPA approved the study, and informed consent was obtained from the patients' guardians prior to enrollment.

The exclusion criteria were congenital infections, congenital malformations, proven sepsis or meningitis, need for intubation in the delivery room, and use of prophylactic surfactant prior to enrollment in the study.

Clinical data included gender, birth weight, GA, Score for Neonatal Acute Physiology Perinatal Extension II (SNAPPE-II) regarding the first 12 hours of life, the type of delivery, and antenatal factors such as the presence of amniorrhexis, preeclampsia, steroid use, and/or chorioamnionitis. The patients were separated into two groups according to oxygen need during their first two days of life: low oxygen (LO), when in MV and requiring ≤30% oxygen, and high oxygen (HO), when in MV and requiring >30% oxygen. The patients received more or less oxygen by the Neonatal Intensive Care Unit (NICU) staff based on their symptoms and signs at the initiation of the treatment, and this was not controlled by the research team.

The neonates were followed from birth to tracheal intubation and the onset of MV; blood samples were collected according to the NICU routine for arterial blood gas analysis, and an additional 500 *μ*L aliquot was obtained for later cytokine analysis. Another sample was collected after two hours of invasive respiratory support. Plasma was frozen at −80°C for laboratory analysis, and the measurement of cytokines was performed using a commercially available multiplex kit (MILLIPLEX® Millipore Corporation). Readings were performed with Luminex 100 technology.

## 3. Statistical Analysis

The Mann–Whitney *U* test compared cytokine levels in both oxygen groups. Wilcoxon's signed-rank test compared the pre- and post-MV interleukin levels. Spearman's test was applied to verify any correlation between oxygen levels and cytokine levels in both MV moments. All analyses were carried out with Statistical Package for Social Sciences (SPSS), version 20.0 (Seattle, USA), and a significance level was established at *p* < 0.05.

## 4. Results

Twenty preterm infants were included: 9 (45%) were males, 7 (35%) were small for gestational age, 16 (80%) were delivered by C-section, and 7 (37%) were delivered by preeclamptic mothers. Only 7 mothers (35%) received a full antenatal steroid course. The mean birth weight was 1921.5 ± 743 grams, and the mean GA was 32.2 ± 3weeks. The median SNAPPE-II score at 12 hours of life was 19 (7–29). The sample collection time for cytokine measurement pre-MV was 9 (3–48) hours of life. Median arterial saturation levels were ≥90% in both oxygen groups and in both moments.

**Figure 1:**
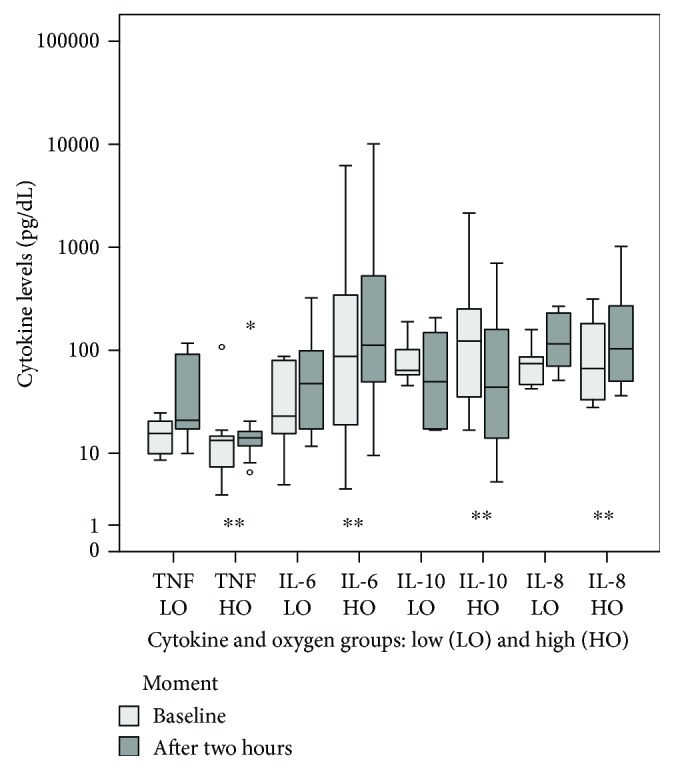
Cytokine levels immediately after the onset of MV and after 2 hours—Wilcoxon's test; ^∗^outlier, ^∗∗^*p* < 0.05.

IL-6, IL-8, and TNF-*α* median plasma levels significantly increased after 2 hours of MV in the high oxygen group (Figure 1). Comparing both oxygen groups, IL-6 levels were higher in the HO group after MV, and IL-10 levels before MV were higher in the HO group than in the LO group (Table 1).

The oxygen levels pre-MV and interleukin levels were compared in both moments. There was a correlation between O_2_ and IL-6 levels collected after two hours of MV (*r* = 0, 48, *p* = 0.03) and a trend of correlation between O_2_ and IL-6 levels collected pre-MV (*r* = 0.4, *p* = 0.08), but there were no correlations regarding the other interleukins or TNF-*α*.

## 5. Discussion

In this pilot study, we demonstrated that even a short period under invasive mechanical ventilation associated with higher oxygen levels may lead to lung inflammation: higher IL-6, IL-8, and TNF-*α* median levels and lower IL-10 median levels were found. This suggests a relationship between VILI and oxygen-induced lung injury.

During hyperoxia, the rupture of the airway epithelium barrier may increase pulmonary permeability, release of inflammatory mediators, and fluid congestion [1], with significant cell infiltration into the immature lung. These cells produce reactive oxygen species prompting pulmonary and airway remodeling, and it has been demonstrated that preterm neonates have elevated levels of proinflammatory mediators in tracheal aspirates and bronchoalveolar lavage fluid [3]. Indeed, all these modifications may occur due to MV per se and not because of high oxygen exposure, although an interaction between both risk factors occurs.

We demonstrated a statistically significant increase in IL-6, IL-8, and TNF-*α* after two hours of invasive mechanical ventilation. In a previous study, which included full-term newborns and late preterm newborns, similar IL-6 levels were obtained after two hours of MV [4], suggesting that lower gestational ages may play a role in IL-6 release. In fact, IL-6 was 6.4 times higher in preterm newborns as compared to full-term newborns at birth, which suggests that inflammatory stress occurs even prior to ventilation, resulting from preterm birth and/or labor [5]. The findings of Leviton et al. [6] also support this theory of a fetal inflammatory response, which is stronger at lower gestational ages.

IL-10 levels pre-MV were higher in the HO group than in the LO group; nevertheless, after MV, these levels had decreased. It is known that the release of anti-inflammatory IL-10 occurs only after the increase of IL-8, notably in immature infants below 30 weeks of GA [7], leaving them more vulnerable to intense proinflammatory response [8]. Pre-MV IL-10 higher levels, however, would suggest an early and efficient response to respiratory distress that required high oxygen, which was reduced after adding MV.

Randomized controlled masked trials enrolling almost 5000 very preterm babies have shown that, once patients are stable, keeping higher oxygen saturation targets (91–95%) until 36 weeks' postmenstrual age showed no differences in the combined outcome of mortality or severe disability compared to lower targets (85–89%); however, mortality before 18 to 24 months was higher in the lower-target group. Recent meta-analyses pooled these results and confirmed that the ideal SpO2 range for extremely low birth weight infants remains unclear [2]. In our sample, the SpO2 range was higher than the recommended in both oxygen groups, but our gestational age range was also higher, involving newborns who were still unstable. Besides that, as extremely preterm newborns are more likely to develop systemic inflammation than other preterm categories, they were not included in this pilot study also to avoid impact on baseline cytokine levels [9].

A trial comparing higher (90–100%) versus lower (21–30%) initial FiO_2_ in the delivery room showed that newborn infants receiving a higher O_2_ load had also higher concentrations of oxidative stress biomarkers and were more predisposed to develop bronchopulmonary dysplasia [10]. A meta-analysis [11] comparing high (>50%) and low (<50%) initial FiO_2_ in patients < 32 weeks of GA found no differences regarding mortality and/or morbidity (from BPD, severe intraventricular hemorrhage, necrotizing enterocolitis, or retinopathy). Conversely, other meta-analyses [12] which included 10 recently published studies showed that mortality was higher in preterm newborns with a high initial FiO_2_ (0.60–1.0) but did not find any differences regarding BPD and intraventricular hemorrhage. The authors therefore concluded that very preterm infants should be initially ventilated with lower FiO_2_ (0.21–0.30) and that they should be titrated according to the neonate's response.

We described a positive correlation between baseline O_2_ levels and IL-6 levels after two hours of MV (*r* = 0.48, *p* = 0.03) and also a trend of correlation regarding pre-MV IL-6 levels (*r* = 0.4, *p* = 0.08). It seems that these preterm infants who needed more oxygen pre-MV presented higher IL-6 levels in both moments, suggesting that they were more injured and sicker at baseline. It is postulated that oxidative stress promotes the expression of cytokines and the inflammatory process during respiratory distress syndrome [13], which is in agreement with our idea of sicker newborns requiring higher oxygen therefore releasing more cytokines. Also, it is well known that proinflammatory mediators such as IL-6 may be increased due to fetal exposure to maternal inflammatory mediators [14]. Thus, it is accepted that previous lung damage makes them more likely to have oxygen-induced injury, leading to inflammation that is not limited to the lung [15].

Despite the small sample studied, data showed that there is a relationship between VILI, proinflammatory cytokines, and oxygen-induced lung injury, but a larger study considering oxidative marker measurements should be carried out. Mechanical ventilation alone, oxygen, or both could have increased inflammatory mediators in this sample, so the causal relationship between them still remains unclear. In view of the trials and meta-analysis described and regarding the pathophysiologic aspects of oxidative stress, one should recommend low oxygen levels enough to keep a safe saturation target, at the most extreme preterm infants.

## Figures and Tables

**Table 1:** Comparing cytokine levels regarding the FiO2 group immediately before the onset of MV and after 2 hours.

	IL-6	IL-8	IL-10	TNF-*α*	IL-1*β*
*Pre-MV*					
FiO2 ≤ 30%	23 (8–71)	56 (34–134)	51 (23–60)	17 (10.5–19)	11 (5.6–23.5)
FiO2> 30%	68 (15–94.5)	80 (32–176)	122 (52–236)	12 (7–13.5)	8 (7–11)
*P* ^1^	0.38	0.68	0.05	0.14	0.38
*After 2 h*					
FiO2 ≤ 30%	21.5 (9–81)	74.5 (56–142)	52 (45.5–169)	17 (10–20)	10 (5–24)
FiO2> 30%	110 (50–490)	179 (51–272)	29 (13–155)	12 (7–13.5)	8.5 (7.5–13)
*P* ^1^	0.05	0.55	0.43	0.61	0.61

^1^Mann–Whitney *U* test.

## Data Availability

No data is available elsewhere regarding this manuscript.

## References

[1] Bhandari V. (2008). Molecular mechanisms of hyperoxia-induced acute lung injury. *Frontiers in Bioscience*.

[2] Manja V., Saugstad O. D., Lakshminrusimha S. (2017). Oxygen saturation targets in preterm infants and outcomes at 18-24 months: a systematic review. *Pediatrics*.

[3] Vogel E. R., Britt R. D., Trinidad M. C. (2015). Perinatal oxygen in the developing lung. *Canadian Journal of Physiology and Pharmacology*.

[4] Bohrer B., Silveira R. C., Neto E. C., Procianoy R. S. (2010). Mechanical ventilation of newborns infant changes in plasma pro- and anti-inflammatory cytokines. *The Journal of Pediatrics*.

[5] Chiesa C., Signore F., Assumma M. (2001). Serial measurements of C-reactive protein and interleukin-6 in the immediate postnatal period: reference intervals and analysis of maternal and perinatal confounders. *Clinical Chemistry*.

[6] Leviton A., Fichorova R., Yamamoto Y. (2011). Inflammation-related proteins in the blood of extremely low gestational age newborns. The contribution of inflammation to the appearance of developmental regulation. *Cytokine*.

[7] Beresford M. W., Shaw N. J. (2002). Detectable IL-8 and IL-10 in bronchoalveolar lavage fluid from preterm infants ventilated for respiratory distress syndrome. *Pediatric Research*.

[8] Jónsson B., Li Y. H., Noack G., Brauner A., Tullus K. (2000). Downregulatory cytokines in tracheobronchial aspirate fluid from infants with chronic lung disease of prematurity. *Acta Paediatrica*.

[9] Goldenberg R. L., Culhane J. F., Iams J. D., Romero R. (2008). Epidemiology and causes of preterm birth. *The Lancet*.

[10] Vento M., Moro M., Escrig R. (2009). Preterm resuscitation with low oxygen causes less oxidative stress, inflammation, and chronic lung disease. *Pediatrics*.

[11] Brown J. V. E., Moe-Byrne T., Harden M., McGuire W. (2012). Lower versus higher oxygen concentration for delivery room stabilisation of preterm neonates: systematic review. *PLoS One*.

[12] Saugstad O. D., Aune D., Aguar M., Kapadia V., Finer N., Vento M. (2014). Systematic review and meta-analysis of optimal initial fraction of oxygen levels in the delivery room at ≤32 weeks. *Acta Paediatrica*.

[13] Gitto E., Reiter R. J., Cordaro S. P. (2004). Oxidative and inflammatory parameters in respiratory distress syndrome of preterm newborns: beneficial effects of melatonin. *American Journal of Perinatology*.

[14] Aversa S., Marseglia L., Manti S. (2016). Ventilation strategies for preventing oxidative stress-induced injury in preterm infants with respiratory disease: an update. *Paediatric Respiratory Reviews*.

[15] Poggi C., Dani C. (2014). Antioxidant strategies and respiratory disease of the preterm newborn: an update. *Oxidative Medicine and Cellular Longevity*.

